# A Multi-Modal Curriculum Teaching Opioid Use Disorder Management in Young Adult Populations

**DOI:** 10.7759/cureus.18499

**Published:** 2021-10-05

**Authors:** Katelyn E Donohue, Mihika Sathe, Samuel Wood, Natalie L Davis, Dara L Farber

**Affiliations:** 1 Internal Medicine and Pediatrics, University of Maryland School of Medicine, Baltimore, USA; 2 Internal Medicine and Pediatrics, Inova Health System, Annandale, USA; 3 Internal Medicine and Pediatrics, University of Maryland Medical Center, Baltimore, USA; 4 Pediatrics, University of Maryland School of Medicine, Baltimore, USA

**Keywords:** adolescent and young adult, opiate use, medical education curriculum, resident education, pediatrics

## Abstract

Background: The use of both prescription and illicit opioids among adolescents and young adults (AYA) is increasing. Barriers to effective treatment of opioid use disorders among AYA range from patients leaving against medical advice to decreased knowledge and experience of providers caring for those with opioid dependence. No formal curricula for residents on AYA opioid use disorder and management have been implemented despite rapidly increasing use in this population.

Objective: To develop a brief curriculum for trainees who encounter AYA that will increase knowledge and skills to treat opioid use in the AYA population.

Methods: Twenty-six pediatric and family medicine interns participated in this pilot study. The multimodal curriculum included standardized patient encounters, case-based learning sessions, didactics, and high-fidelity simulations. The curriculum encompasses five individual sessions, each with a different theme: motivational interviewing, naloxone administration, opioid withdrawal medications, complex overdoses, and infectious complications of intravenous drug use. A pre-survey was administered prior to the curriculum and a post-survey was administered at the conclusion to assess its effectiveness in improving knowledge for this specific population and increasing comfort levels providing medical interventions in AYA patients with opioid use disorders.

Results: Trainee comfort levels increased significantly in all four domains as measured by the average Likert scale, including interviewing AYA about opioid use (2.5 (standard deviation (SD) 1.2) to 4 (SD 0.9), p<0.0001)), prescribing medication for opioid use disorder (1.3 (SD 0.5) to 2.8 (SD 1.3), p<0.0001)), treating acute opioid overdose (1.5 (SD 0.8) to 3.7 (SD 0.9), p<0.0001)), and treating infectious complications of intravenous drug use (1.7 (SD 0.8) to 3 (SD 1.1), p <0.0001)). The Chi-square test showed similarly significant increases in comfort levels.

Conclusions: Early trainees who provide care to young adults benefit from opioid education specific to this population. Participants described increased knowledge and comfort in interviewing and treating this vulnerable patient group.

## Introduction

Use of both prescription and illicit opioids has increased among adolescents and young adults (AYA) in the past two decades [1]. Opioid use is associated with myriad health complications including infection risk, addiction, worsening mental health issues, and death [2,3]. Barriers to effective treatment of opioid use disorder among AYA include higher rates of attrition from inpatient detoxification programs and decreased knowledge and experience of providers caring for those with opioid dependence [3,4]. Although trainees in residency programs are often first-line providers for opioid misuse and overdose, the Accreditation Council for Graduate Medical Education (ACGME) currently does not require specific education or curricular activities regarding opioid use disorder for pediatrics or family medicine residency training [5,6]. 

Pediatric providers are increasingly taking care of patients with preventable opioid exposure and overdoses in children’s hospitals across the nation [7]. Despite this increased exposure, pediatric residents are often uncomfortable with counseling patients or treating acute opioid overdose [8]. No formal curricula for pediatric residents on AYA opioid use disorders and management have been implemented in a residency program as noted via literature searches on PubMed and the MedEdPortal. The use of motivational interviewing has been reported to have a positive effect on adolescent substance abuse and has been established as an effective strategy for AYA, regarding both lifestyle measures and substance abuse [9-11]. General principles of addiction, substance abuse screening, and motivational interviewing have been implemented in adolescent medicine fellowships with pediatric resident rotators, but with little emphasis on acute management or detoxification [12]. 

Didactic and skills sessions for internal medicine, family medicine, and emergency medicine residents have shown that specific training, such as naloxone training, is effective in improving overall comfort in caring for adults with opioid use disorder and change in clinical practice [13-16]. Given the success of dedicated curricula for trainees of other specialties regarding adult opioid use disorder, we developed a clinically translatable curriculum for pediatric and family medicine residents to specifically identify and treat AYA opioid dependence and withdrawal in pediatric patients, with hopes of improving resident comfort treating opioid use disorder and addiction in clinical practice. 

## Materials and methods

Participant characteristics and approval

Eleven family medicine interns and 15 categorical pediatric interns from the University of Maryland Medical Center participated in a course consisting of five lessons on opioid dependence, withdrawal, and management in AYA. Family medicine interns participated in five sessions over three days during their orientation in June of 2019. Categorical pediatrics interns participated in five sessions over two days in the fall of 2019. This study was deemed as exempt from IRB review by the University of Maryland Institutional Review Board under 45 CFR 46.101(b) based on Category (2).

Curriculum design 

The curriculum was designed as a collaboration between board-certified providers in internal medicine and pediatrics with experience in curriculum design, addiction psychiatry specialists, and physicians with specific experience in the management of opioid use in young adults. It was developed and administered as a series of five sessions, with each session focusing on a different component of AYA opioid knowledge and containing both a didactic portion and an interactive skills session. For the purposes of our study, we defined AYA as patients <22 years of age. 

Session 1 

In the introductory lesson, learners were educated on how to sensitively approach the topic of addiction and motivational interviewing during a standardized patient ambulatory clinic encounter adapted from the National Institute of Drug Abuse (NIDA) Medical School and Residency Curriculum Resources on Drug Abuse and Addiction [17]. The curriculum includes techniques for discussing sensitive topics, screening methodologies, and engagement in primary care settings for adolescent patients [17]. We started with a didactic session on reviewing interviewing techniques followed by learners interviewing a standardized patient in a simulated clinic session. Learners took turns in the simulated clinic sessions. The interviews were observed in real time by curriculum leaders and other learners. There was a formal debriefing session for learners with curriculum leaders and the standardized patient at the conclusion of the session.

Session 2 

Learners participated in didactics on opioid withdrawal and treatment followed by two case-based learning sessions in small groups requiring them to identify acute opioid withdrawal using validated scores such as the Clinical Opiate Withdrawal Scale (COWS) [18] and implement different treatment options, including buprenorphine-naloxone and methadone. Each small group of learners was asked to proceed through the cases and questions as a team. This was followed by a large group discussion in which correct answers were revealed. Small group answers were not scored.

Session 3 

Learners participated in a workshop reviewing the properties of naloxone. A didactic session was followed by four hands-on stations where learners practiced administering rescue naloxone doses through intramuscular (IM), nebulized, intravenous (IV), and intranasal (IN) models, including the Evzio® Naloxone auto-injector (Kaleo Inc., Richmond, Virginia), a hand-held single-dose automatic injector device that provides verbal instructions when activated on how to deliver its dose of intramuscular naloxone.

Session 4 

Learners participated in a lecture on the management of opioid overdose, followed by high-fidelity simulation scenarios on managing acute opioid overdose. Simulations occurred in a variety of settings (clinic, emergency department) and required learners to demonstrate both simple administration of naloxone as well as more advanced skills, such as initiating a naloxone infusion. 

*Session 5* 

During the fifth session, learners participated in high-fidelity simulations designed to familiarize learners with complex opioid overdose and management of infectious complications of intravenous drug use (IVDU) such as cellulitis, endocarditis, and HIV. Prior to the simulation sessions, learners participated in a didactic session on the infectious complications of IVDU.

Assessment 

Participants received an anonymous pre-curriculum survey prior to the curriculum and an identical post-curriculum survey after completion. The survey assessed prior experience in treating opioid use disorders and utilized a Likert scale to assess resident comfort with “1” corresponding with “not at all comfortable” and “5” corresponding with “very comfortable”. Categorical data were compared using the chi-square test and mean scores regarding comfort levels as defined on the Likert scale were compared using the two-tailed t-test. All data were analyzed using SAS® 9.4 (2013, SAS Institute Inc., Cary, North Carolina).

## Results

Baseline experiences 

In the 2019-20 academic year, 15 categorical pediatric (57.7%) and 11 family medicine (42.3%) interns participated in the curriculum. All subjects (n=26) completed pre-curriculum surveys. Twenty-five subjects completed the post-curriculum survey.

Subjects were surveyed about their baseline clinical experiences regarding opioid use disorder in AYA patients (under the age of 22). The majority had encountered few (<5) AYA patients with opioid dependence (n = 18, 72%) and an even greater number had only treated acute withdrawal in few (<5) AYA patients (n=22, 84.6%). Only two participants (7.7%) had ever prescribed medication-assisted treatment (MAT) for opioid use disorder in >5 adult patients and only one had done so in >5 AYA patients. The low number of encounters with AYA patients with opioid use disorder was expected as this curriculum was administered early during the learner's intern year of residency training.

Resident knowledge 

Participants were asked about knowledge and comfort in four areas: 1) interviewing AYA about opioid use, 2) prescribing MAT such as buprenorphine-naloxone or methadone, 3) treating acute opioid overdose in the hospital setting, and 4) treating infectious sequelae of IVDU. 

Mean Likert scores on the post-curriculum survey were significantly higher in comparison to the pre-curriculum survey in all four areas assessed (Table 1). The overall average Likert score among participants regarding the comfort of interviewing AYA with opioid use increased from 2.5 (SD 1.2) to 4 (SD 0.9) after implementation of the curriculum (p-value <0.0001). The mean scores in comfort levels of prescribing MAT increased from 1.3 (SD 0.5) to 2.8 (SD 1.3) after the curriculum among the group (p-value <0.0001). The pre-curriculum mean comfort level among participants was 1.5 (SD 0.8) when asked about the comfort level of administering naloxone and rose to 3.7 (SD 0.9) after implementation of the curriculum (p-value <0.0001). Resident comfort in caring for infectious sequelae of IVDU increased on average among the group from a pre-curriculum mean of 1.7 (SD 0.8) to a post-curriculum mean of 3 (SD 1.1) (p-value <0.0001).

**Table 1:** Comparison of Participant Comfort Before and After Educational Curriculum. Participant comfort was defined on a Likert scale with 1= not at all comfortable to 5= very comfortable. AYA: adolescent and young adult; IVDU: intravenous drug use

	Pre -Curriculum Survey Mean (SD)	Post-Curriculum Survey Mean (SD)	p-value
Interviewing AYA about opioid use	2.5 (1.2)	4 (0.9)	< .0001
Prescribing maintenance treatment	1.3 (0.5)	2.8 (1.3)	< .0001
Administering naloxone in the ER/inpatient setting	1.5 (0.8)	3.7 (0.9)	< .0001
Treating for infectious sequalae of IVDU	1.7 (0.8)	3 (1.1)	< .0001

When comparing categorical pediatrics and family medicine trainees, there were no significant differences on the pre-test, indicating similar levels of comfort before the curriculum regardless of the training program. On the post-curriculum survey, both groups showed statistically significant improvement in comfort in all four areas after the curriculum as noted above; however, the family medicine trainees had significantly higher comfort in the post-survey than the categorical pediatrics residents in three areas: interviewing YA patients (p-value =0.042), administering naloxone (p-value =0.042), and treating infectious sequelae of IVDU (p-value =0.042).

**Figure 1:**
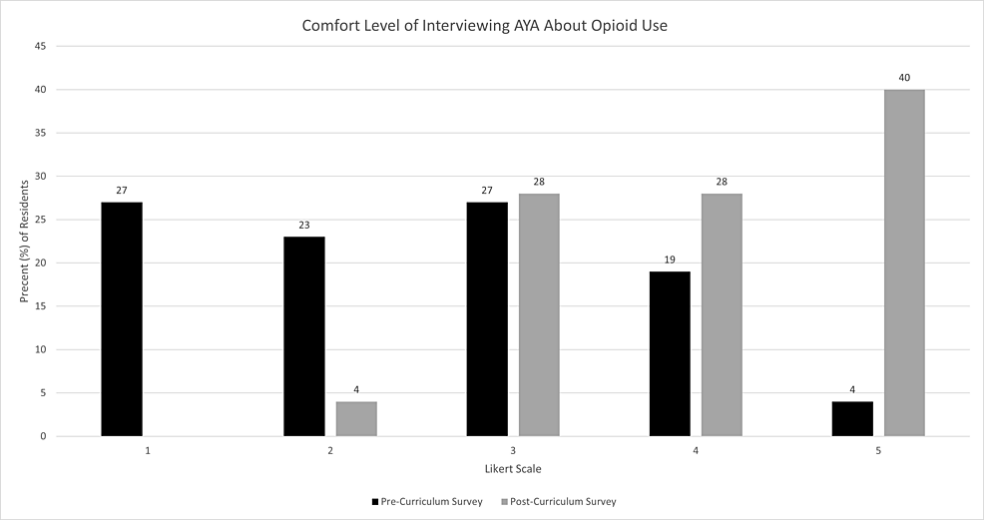
Participant comfort level of interviewing AYA about opioid use before and after educational curriculum.

**Figure 2:**
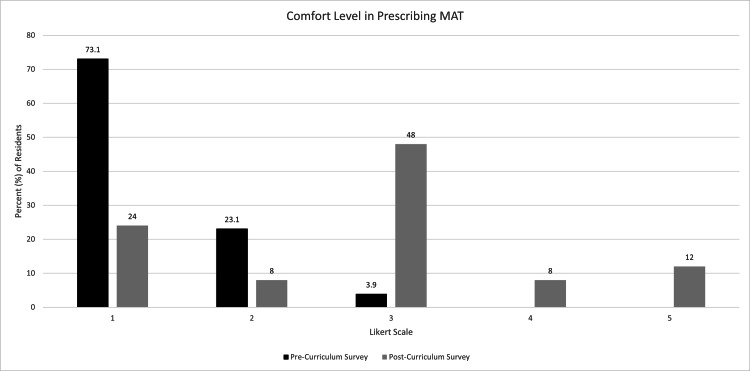
Participant comfort level of prescribing MAT for opioid use disorder before and after educational curriculum.

**Figure 3:**
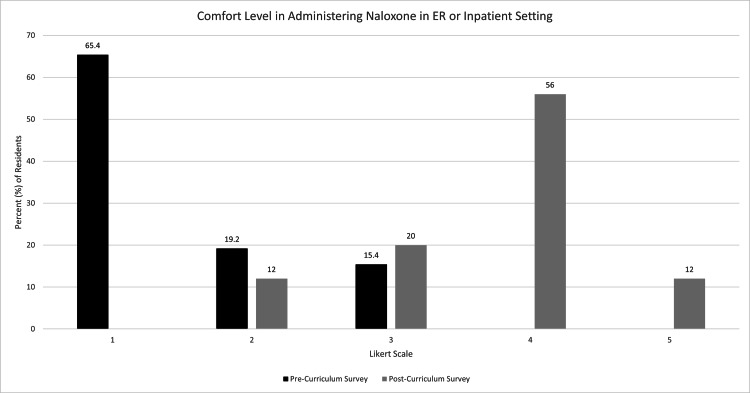
Participant comfort level in administering naloxone before and after educational curriculum.

**Figure 4:**
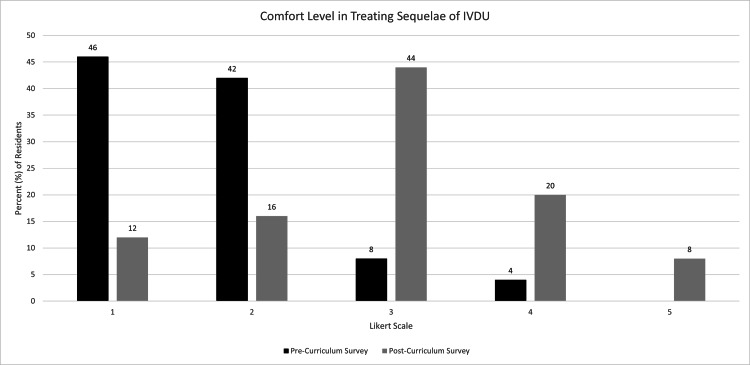
Participant comfort level in treating infectious sequelae of IVDU before and after educational curriculum.

Comfort level in all four of these measures by the distribution of Likert scores was also compared via a chi-square test, which showed significance for all four measures. Prior to initiation of the curriculum, one participant (4%) indicated a “very comfortable” level in interviewing AYA about opioid use while 10 participants (40%) indicated a “very comfortable” level afterward (p-value = 0.0011) (Figure 1). No participants indicated a complete lack of comfort in interviewing AYA after the curriculum. For comfort prescribing MAT to AYA, 19 individuals (73.1%) indicated they were not at all comfortable on the pre-curriculum survey, while only six individuals (24%) indicated as such on the post-curriculum survey (p-value = 0.0001) (Figure 2). After the curriculum, no respondents indicated a complete lack of comfort with prescribing naloxone compared to 17 respondents (65.4%) initially (p-value < 0.0001) (Figure 3). Post-curriculum Likert scores also increased for the comfort level of treating infectious sequelae of IVDU (p-value = 0.0006) (Figure 4).

Residents were also assessed on the comfort level in administering naloxone to bystanders who had overdosed outside of the hospital. Subjects answered “not at all comfortable” or “comfortable”. Pre-curriculum, 18 out of the 26 residents (69.2%) answered that they would not be comfortable in administering naloxone outside the hospital. Post-curriculum, all 25 residents (100%) who answered the survey indicated that they had some level of comfort in out-of-hospital naloxone administration (p-value <.0001).

## Discussion

This pilot study demonstrates that implementation of a dedicated curriculum in caring for AYA with opioid use disorder increases comfort level of early-level resident trainees in treating this unique patient population. Comfort levels in four major areas, including interviewing AYA about opioid use, prescribing MAT, treating acute opioid overdose, and treating infectious complications of IVDU, increased significantly after participants completed the curriculum.

Although this study was based in an urban academic center located in Maryland, where deaths from opioid use are second in the nation [19], we believe that it is generalizable to most (if not all) residency training programs. There is strong evidence that the opioid epidemic remains a nationwide issue as demonstrated by recent joint statements from several physician-affiliated organizations, including the American Academy of Family Medicine and the American Academy of Pediatrics [20]. This curriculum addresses several points in the joint statement, including increasing overall provider comfort, reducing stigma through education, and disseminating knowledge of evidence-based treatments for opioid use disorder. After participating in the curriculum, several learners expressed interest in pursuing further certification in obtaining buprenorphine waiver training.

This study has several limitations that could be addressed in future iterations. The novel curriculum was piloted at a single urban academic center among a small number of resident trainees in their first year of training and is therefore limited in scope. Though mean comfort levels increased from pre- to post-curriculum, the effectiveness of the curriculum on an individual level could not be assessed. However, we suspect that individual skill levels increased in at least some facets of care including interviewing AYA about opioid use and treating opioid overdose in a hospital setting, as no residents answered “not at all comfortable” after the educational intervention. The post-curriculum differences in comfort level between family medicine and categorical pediatrics trainees may be related to overall increased exposure in family medicine trainees to substance use disorders during their intern year of training. This further highlights the need for training specific to pediatrics trainees, who frequently encounter AYA patients, as well as objective measurements of knowledge to differentiate between a comfort gap and a true knowledge gap. We note that comfort levels as defined by the Likert scale are subjective measures and not validated knowledge-based assessments. Future iterations of this curriculum may benefit from the incorporation of objective, validated knowledge-based assessments, comparison to non-intervention intern trainees, and knowledge assessment of upper-level residents participating in a formal curriculum versus standard education, as well as linking pre-curriculum and post-curriculum responses to evaluate the curriculum at the individual level.

The curriculum itself utilized a combination of didactic and hands-on or simulation-based exercises. Post-curriculum, all residents reported increased comfort in interviewing AYA and administering naloxone, which was part of the simulated and hands-on formats, respectively. Withdrawal identification and MAT were taught via a case-based learning session. There was also an increase in the number of residents who indicated that they were comfortable prescribing MAT after the curriculum, but these improvements were more modest. This highlights the role of the educational format in influencing the effectiveness of this curriculum and is an area of interest for future studies.

We aim to share this educational curriculum that may help standardize the approach to caring for AYA with opioid use disorder among resident trainees. The overarching goal of these educational interventions is to help address the opioid epidemic by increasing provider comfort and provide better care for vulnerable AYA patients.

## Conclusions

This pilot study demonstrated that implementation of a curriculum dedicated to care of AYA patients with opioid use disorder increased comfort level of early trainees in treating this vulnerable patient population. This new curriculum can be implemented in pediatric residency programs, as well as other training programs that treat young adult patients such as family medicine or internal medicine-pediatrics. Expansion of this curriculum to other programs and institutions who train pediatric providers should be considered as the opioid crisis shows no signs of abating and pediatric providers will increasingly encounter opioid use issues in their inpatient and outpatient practices.
